# Hospitals as complex adaptive systems: A case study of factors influencing priority setting practices at the hospital level in Kenya

**DOI:** 10.1016/j.socscimed.2016.12.026

**Published:** 2017-02

**Authors:** Edwine W. Barasa, Sassy Molyneux, Mike English, Susan Cleary

**Affiliations:** a KEMRI Centre for Geographic Medicine Research – Coast, and Wellcome Trust Research Programme, Nairobi, Kenya; b Health Economics Unit, University of Cape Town, Cape Town, South Africa; c Centre for Tropical Medicine, University of Oxford, Oxford, UK; d Department of Pediatrics, University of Oxford, Oxford, UK

**Keywords:** Priority setting, Complex adaptive systems, Systems thinking, Hospitals, Kenya

## Abstract

There is a dearth of literature on priority setting and resource allocation (PSRA) practices in hospitals, particularly in low and middle income countries (LMICs). Using a case study approach, we examined PSRA practices in 2 public hospitals in coastal Kenya. We collected data through a combination of in-depth interviews of national level policy makers, hospital managers, and frontline practitioners in the case study hospitals (n = 72), review of documents such as hospital plans and budgets, minutes of meetings and accounting records, and non-participant observations of PSRA practices in case study hospitals over a period of 7 months. In this paper, we apply complex adaptive system (CAS) theory to examine the factors that influence PSRA practices. We found that PSRA practices in the case hospitals were influenced by, 1) inadequate financing level and poorly designed financing arrangements, 2) limited hospital autonomy and decision space, and 3) inadequate management and leadership capacity in the hospital. The case study hospitals exhibited properties of complex adaptive systems (CASs) that exist in a dynamic state with multiple interacting agents. Weaknesses in system ‘hardware’ (resource scarcity) and ‘software’ (including PSRA guidelines that reduced hospitals decision space, and poor leadership skills) led to the emergence of undesired properties. The capacity of hospitals to set priorities should be improved across these interacting aspects of the hospital organizational system. Interventions should however recognize that hospitals are CAS. Rather than rectifying isolated aspects of the system, they should endeavor to create conditions for productive emergence.

## Highlights

• Priority setting practices reveal the complex adaptive systems (CAS) nature of hospitals.
• Hospital priority setting is influenced by deficiencies in both system hardware and software.
• Intervening in CAS requires fostering productive emergence, not simple, mechanistic solutions.

## 1 Background

Given that healthcare demand outstrips available resources, priority setting and resource allocation (PSRA) has been considered a key determinant of health system performance (Martin, 2007). This is particularly true for low and middle income countries (LMICs) where the gap between healthcare needs and health system resources is wide (Kapiriri and Martin, 2007). Whereas PSRA occurs at every level of the health system, research has mainly focused on macro (national) and micro (patient) level processes and rarely on the meso (regional and/or health facility) level, particularly hospitals (Barasa et al., 2015). This is surprising given the critical role that hospitals play in the delivery of healthcare services and the relatively high costs of hospital level care. For example, in Kenya public hospitals are estimated to consume over 50% of the public sector healthcare budget (Glenngård and Maina, 2007). Further, a recent literature review on hospital level PRSA practices found that most studies focused on developed country settings, and on tertiary level, mostly teaching hospitals (Barasa et al., 2015). These hospitals are often quasi-autonomous institutions whose operations, organizational structures and processes, resources and target users are very different from lower level hospitals (Barasa et al., 2015). There is therefore a gap in understanding how smaller, first-referral hospitals conduct PSRA, especially in LMICs. This paper presents case study research of priority setting practices in public first level referral hospitals (known as county hospitals) in Kenya.

At the time of doing this study, the Kenyan health sector was significantly decentralized with the district health system as the focal point. The public healthcare delivery system is organized into four tiers, namely community services, primary care, 1st level referral services in county hospitals (formally district), and national referral services. Little is known on how the Kenyan health sector sets its priorities. At the macro level, it has been reported that priority setting is ad hoc, rather than systematic, without explicit priority setting criteria (Ndavi et al., 2009). Priority setting at the district level (now counties), relies heavily on strict guidelines from the national level and thus limiting local input into decision making (Bukachi et al., 2014). There are no official guidelines in place on how priority setting should be conducted at the county hospital level. There is also no evidence/literature on how the priority setting process is actually carried out within hospitals in Kenya.

We conducted a qualitative case study in county hospitals in Kenya with the aim of identifying the factors that influence their priority setting practices, examine how these factors influence these processes, and to draw lessons and recommendations on how priority setting practices in these hospitals and similar settings could be improved.

## 2 Methods

This paper is based on the analysis of data collected as part of a wider study conducted to describe and evaluate PSRA practices in first referral public hospitals (county hospitals) in Kenya. A case study approach was employed given its appropriateness in examining complex social phenomena (Yin, 2003). Two hospital cases were selected purposefully guided by the following criteria: 1) 1st level referral hospitals that were designated as county hospitals; 2) hospitals with a high local resource level and those with a low local resource level; 3) hospitals which had prior relationships our research institution. This last criterion was important because the subject of priority setting was likely to be viewed as political and sensitive. By identifying hospitals with prior contact/relationship or linkage with our institution, we aimed to minimize trust concerns. The selection of hospital cases aimed to identify hospitals that were different from each other and to ensure depth in information, as opposed to aiming for representativeness of all county hospitals in Kenya. To maintain confidentiality and minimize the potential identification and possible victimization of study participants, the hospitals selected for the study will only be identified as Hospital A and B. Table 1 outlines some significant characteristics of the case study hospitals.

Within each case study hospital, we selected and conducted in-depth study on 3 PSRA activities to act as tracers for priority setting practices in hospitals. We used three criteria to select priority-setting activities: 1) availability and reliability of information; 2) a clearly defined beginning and end to the activity; and 3) full consent to examine the priority-setting case from the hospital. Based on these criteria, we selected the following three PSRA activities: 1) the hospital budgeting and annual work planning process; 2) medicine selection decisions in the hospital; and 3) nursing allocation to departments in the hospital.

Data were collected over a 7 month period between 2012 and 2013. Data collection was carried out by the first author (EB), through a combination of in-depth interviews of hospital managers and frontline workers, review of relevant documents including hospital plans, budgets, minutes of meetings, and non-participant observations. The selection of participants for interviews was purposive with the aim of selecting those who had an in-depth knowledge and experience of the identified PSRA activities. This included senior managers, middle level hospital managers and frontline practitioners. In total, 72 participants were interviewed; 35 from Hospital A, 32 from Hospital B, and 5 from the national level (Table 2). More middle level managers, compared to senior managers were interviewed because most managers in the hospital were middle level managers. The hospitals had only between 3 and 4 senior managers, all of which were interviewed. Senior managers included the medical superintendents (hospital chief executive officers) who were always clinicians, the hospital nursing officer in charge who were nurses by profession, hospital administrators who had public administration training, and hospital accountants. Middle level managers were in charge of hospital departments and were a mix of healthcare professionals and non-healthcare professionals.

### 2.1 Application of theory

**Fig. 1:**
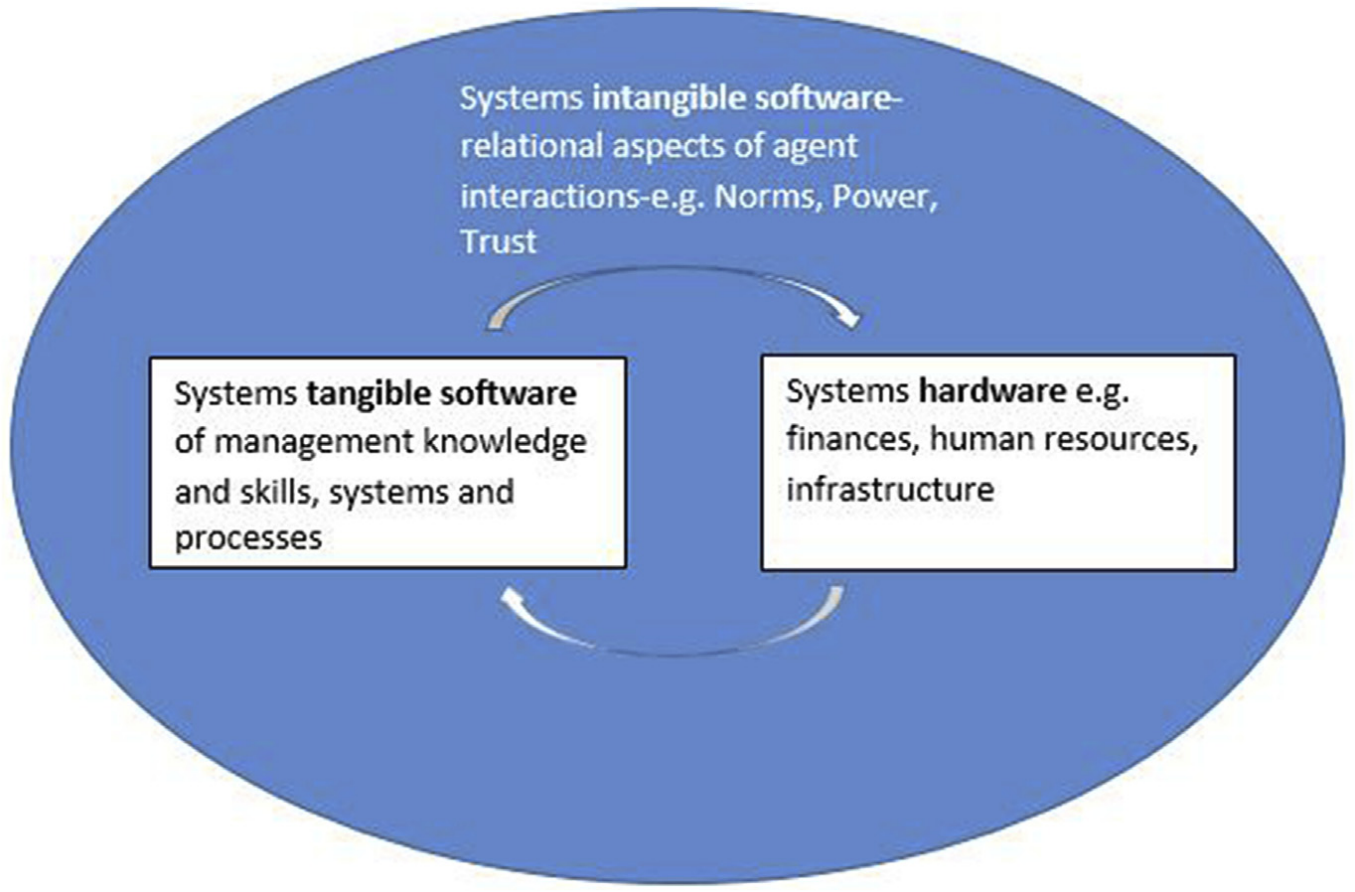
Framework for organization capacity (Elloker et al., 2012).

To unpack the factors that interact with and influence PSRA practices in the case study hospitals, we apply a framework of organizational capacity developed by Elloker et al. (2012), which in turn draws on an understanding of capacity from Aragón and Giles Macedo (2010). In the Elloker et al. (2012) framework, healthcare organizations are seen to be composed of *hardware* and *software* components (Fig. 1). Hardware includes components such as infrastructure, technology and resources. System software includes the *tangible software* of management knowledge and skills, and organizational systems and procedures as well as the *intangible software* of values and norms, relationships and power. While this framework provides a means of characterizing the nature of system components, it does not illuminate on the interactions between these components. To examine and explicate the interactions between these hardware/software capacities of the system, we apply Complex Adaptive Systems (CAS) theory. Rather than focusing on simple cause and effect, the CAS approach sees healthcare and other systems as composed of multiple interconnected components with agents whose interactions and processes are dynamic, simultaneously affecting and shaping the system (Begun et al., 2003, Health Foundation, 2010). While a number of properties of CAS have been proposed, four particular characteristics appear to be agreed upon by most complexity theorists (Begun et al., 2003, Marion and Bacon, 2000). First, CAS are characterized by *self - organization and emergence.* Self- organization is the process by which system components and agents mutually adjust their behaviors in ways needed to cope with changing internal and external environmental demands (Lindberg et al., 2008). Self-organization of the system leads *emergence*, the appearance of outcomes such as new structures, patterns of behavior or processes that are unintended and unpredictable from the components that created them (Zimmerman, 2010). Non-additive and unintended/unanticipated patterns of behavior emerge from these interactions such that the behavior of the resulting whole is different from the sum of individual behaviors (Begun et al., 2003, Marion and Bacon, 2000). Second, CAS exhibit nonlinear behavior, or behavior that is unpredictably related to input (Begun et al., 2003, Marion and Bacon, 2000). Small changes in variables can have small impacts at some times, and large impacts under other conditions (Begun et al., 2003, Marion and Bacon, 2000). Third, CAS exhibit behavior that is on the border of predictability and unpredictability; hence complex dynamics are sometimes referred to as operating on the edge of chaos (Marion and Bacon, 2000, Marion, 1999). As a result, a fourth property of CAS is that they are resilient or robust (Begun et al., 2003, Marion and Bacon, 2000). The amazing resilience of CAS is achieved in part through the range of coupling patterns they exhibit, from loose to tight. Coupling refers to the strength of relationships among units in a system (Marion, 1999). A system is tightly coupled if there is a strong relationship among its units and vice versa (Marion, 1999). In a tightly coupled system, the behavior in one unit strongly affects other units, while in a loosely coupled system, activity or changes in one unit weakly affects other units (Marion, 1999, Weick, 1976). These patterns, together with complex interactions between system components, provide multiple and creative paths for action and enable organizations to adapt to and survive a wide range of environmental conditions (Marion and Bacon, 2000, Marion, 1999).

### 2.2 Data analysis

Transcribed data were imported into NVIVO 10 for coding and analyzed using a framework (thematic) approach (Pope et al., 2000). Data analysis was led by the first author (EB), with support from all authors. Data were analyzed using a modified framework (thematic) approach. This approach was adopted so as to provide findings and interpretations that are relevant to policy and also to provide pragmatic recommendations. However the approach was modified to include an initial open coding step to allow for emergence of important themes which might not have been captured in the study's theoretical frameworks. Coded and charted data were critically examined under each thematic category. Interpretation of the data entailed identifying key concepts and explaining relationships between these key concepts. Also, it entailed explaining relationships between the data and theoretical assumptions and identifying messages that are relevant to policy makers. Rigor and trustworthiness were enhanced by a combination of 1) use of theory (Gilson, 2012), 2) use of multiple rather than single case study, 3) prolonged engagement (7 months) during data collection (Yin, 1999), 4) methodological triangulation.

## 3 Results

**Fig. 2:**
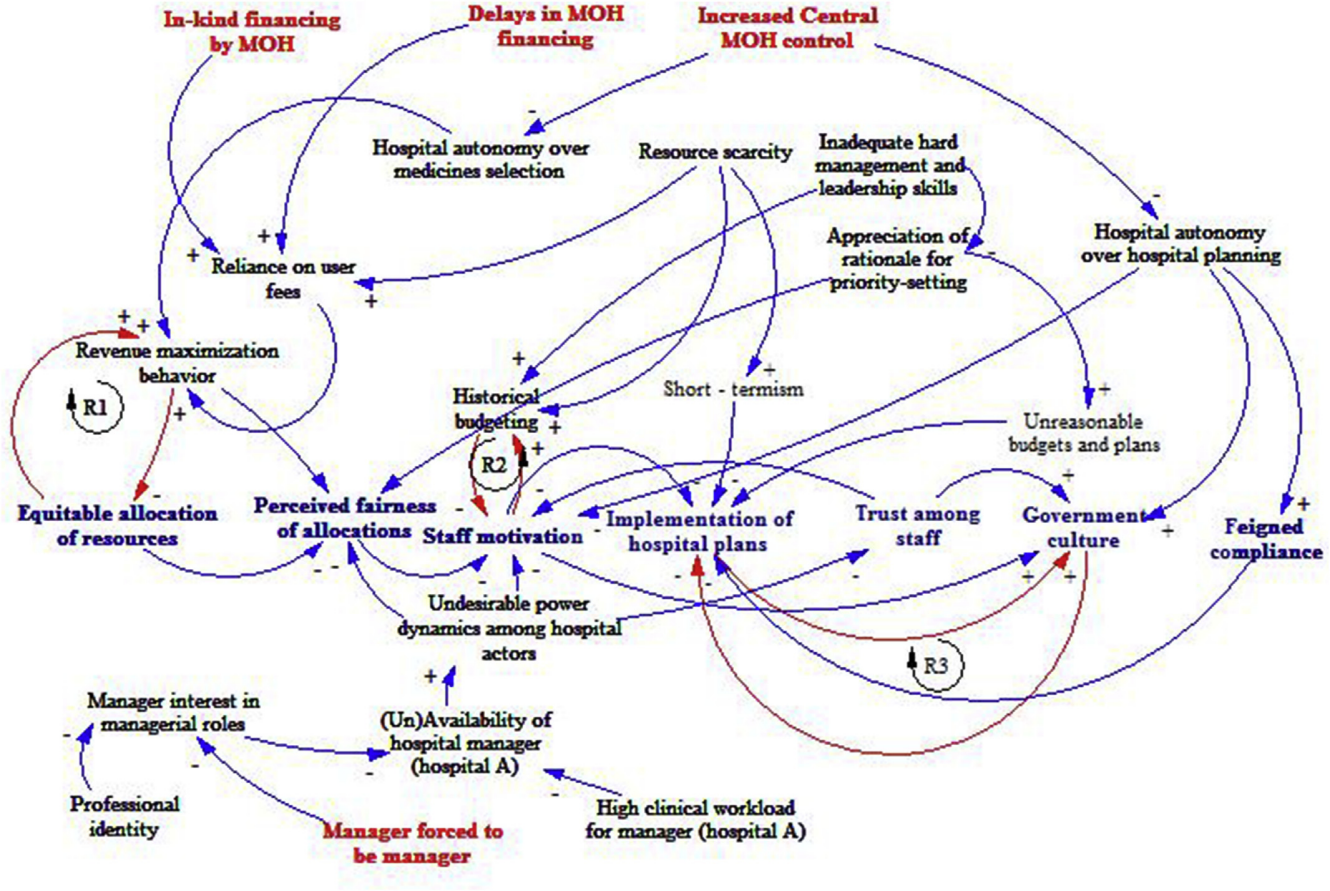
Causal loop diagram of factors affecting priority setting processes in case hospitals.

Even though our criteria for case hospital selection assumed that priority setting practices are likely to vary across hospitals based on differences in their level of resources, our findings did not support this assumption. We found that a number of factors interacted with PSRA processes in the case hospitals including 1) hospital financing 2) hospital autonomy and decision space, and 3) hospital management and leadership. Even though we present these factors separately, there are significant interactions among them, consistent with CAS, in practice. Fig. 2 presents a causal loop diagram of the interaction between internal and external factors to influence priority setting practices in the case study hospitals. The red text, represent factors external to the hospital. The black text represent internal hospital factors, while blue text represent the features of the hospital priority setting processes as a result of the interactions of both the external and internal factors. Arrows indicate causation. Where causal arrows are accompanied by a positive (+) sign, it means the causing factor increases the outcome and vice versa. Red colored arrows signify feedback loops, while the letter R means that the feedback loop is reinforcing rather than self-regulating. The sections below will present a more detailed descriptions of these interactions.

### 3.1 Inadequate financing level and poorly designed financing arrangements

The case study hospitals, as with all other public hospitals in Kenya, received funding from three main sources. First, the central MOH funded hospitals by 1) recruiting and remunerating professional human resources such as doctors, nurses and administrative staff, 2) procuring and supplying essential medical supplies (pharmaceuticals, non-pharmaceuticals and medical equipment) to the hospitals, and 3) allocating funds to hospitals through the hospital management services fund (HMSF). The HMSF was a central MOH fund, supported by general tax allocations from treasury, whose purpose was to provide direct financing to public hospitals for their recurrent and capital budgets. HMSF monetary allocations were, on paper, supposed to be disbursed to hospitals’ bank accounts quarterly. Hospitals were expected to budget and request for an authority to incur expenditure (AIE) from the central MOH before utilizing these funds. Pharmaceuticals and non-pharmaceutical supplies were to be ordered by hospitals and supplied by the Kenya Medical Supplies Agency (KEMSA), a public corporation responsible for the procurement and distribution of pharmaceuticals to public healthcare facilities, every two months. On paper, human resources were allocated as and when needed, depending on availability and guided by the MOH staffing norms for health facilities. Second, the MOH also adopted a cost-sharing policy that required hospitals to charge user fees on services offered to patients (MSH, 2001). User fee revenues were intended to supplement central MOH financing and were referred to as the facility improvement fund (FIF). Third, hospitals also benefited from donations from non-governmental organizations and charitable organizations.

### 3.2 Hospital financing in practice and its influence on PSRA in case study hospitals

**Fig. 3:**
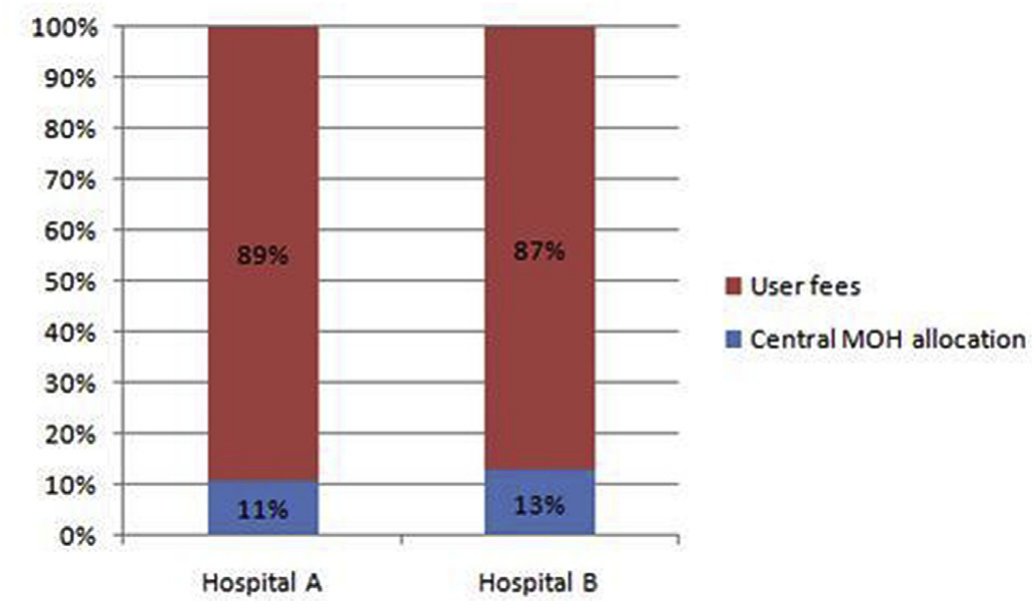
Sources of cash resources for case study hospitals.

Three features of hospital financing interacted to influence the PSRA process (Fig. 3). First, whereas the central MOH was the major source of financing in both case study hospitals, this funding was mostly in the form of human resources and essential medical supplies with only a small proportion being direct monetary allocations. This characteristic of the MOH policy of hospital financing, a weakness of the hospital tangible software of systems and processes, meant that in both case study hospitals, user fees accounted for a higher proportion of cash budgets.

Second, both hospitals were severely underfunded and experienced significant resource scarcity, a weakness in the hospital systems hardware. Chronic underfunding was feature of the Kenyan public sector due to resource scarcity. This was compounded by the cost-sharing policy that required hospitals to generate revenues by collecting user fees from a predominantly poor population. This resource scarcity was reflected across each of the priority setting tracer activities.

“Resources are a challenge to us. If you look at the money we collect and look at the budget requests you will see that we have huge gaps, almost 50%. It is a challenge for us to pay for services, to buy drugs and non-pharmaceuticals and to pay casual workers. We are always in financial problems.” Senior manager, Hospital A

Third, bureaucratic inefficiencies meant that MOH financing systems and processes were unreliable and unpredictable, a weakness in this important tangible software element of hospital functioning. This was reflected in the government budgetary allocation to hospitals and supply of pharmaceuticals and non-pharmaceuticals. While the hospitals were expected to receive quarterly disbursements of HMSF funds, and a bi-monthly supply of essential supplies, both hospitals experienced significant delays.

“Usually, the [HMSF] allocation is done quarterly, but usually it's very late. You can get an AIE but there are no funds in the account. So we cannot depend on the ministry [of health] allocation. Like now [July] we are supposed to get the first quarter allocation but we will probably get it in September when the quarter is almost ending.” Senior manager, Hospital B

The fact that a significant proportion of their major source of financing (the central MOH) was in kind meant that hospitals had little flexibility from this form of financing. Further, the resource scarcity that the hospitals faced and delays experienced with all forms of MOH financing meant that they had a heavy reliance on user fees collected locally since this was readily accessible.

“FIF [Facility improvement fund - this refers to user fee revenues] is very important, because if FIF is stopped today the hospital will shut down. This is because even though the government gives us some drugs and some non-pharmaceuticals, we still have to use cost sharing money to buy most of them. Even the food for patients is bought using cost sharing money … Water bills, electricity bills are all paid with cost sharing money.” Senior manager, Hospital A

Consistent with CAS features, these weaknesses in hospital system hardware and tangible software led the case hospital systems to *self–organize* in response to multiple environment pressures by adopting strategies that maximized their user fee revenues. An emergent property of the hospital was *revenue-maximization behavior*. The hospital management in both case study hospitals adopted the use of the *revenue generating potential* of departments and services as the main PSRA criteria. Managers in both hospitals, who are agents in the hospital system, had an incentive to favor departments that generated more revenues over departments that generated less user fee revenues in their budget allocations. The revenue maximization behavior resulted in inequitable allocation of resources across departments and service areas. Consistent with CAS, a feedback loop linked revenue-maximization behavior with inequitable allocation of resources. Given that the resource generation potential of a service area was, among others, dependent on service provision, a reinforcing feedback loop (R1 in the CLD) was created that operated to worsen the inequity situation. The more a department was underfunded, the less resources it generated and the less resources it attracted in subsequent allocations. On the other hand, the more a department was funded, the more resources they generated, and the more resources they attracted in subsequent allocations. While hospital managers justified this behavior as necessary for the hospital survival, the use of revenue maximization led to undesirable consequences, including decision-making that went against government objectives to increase facility access to special interest groups. For example, the MOH, in an endeavor to increase access to treatment to children under five had made services to this population group free in all public health facilities in Kenya. However with revenue generation as a criterion for allocation of funds across departments, this free care policy led to pediatric departments in the case study hospitals being perceived as low income generators and hence of lower priority.

“Since I am allocated a small budget I only procure medicines that I can sell, I cannot buy medicines for children under 5 years because they don't pay for services.” Senior manager, Hospital A

The preference for high revenue generating departments led to perceptions of unfairness in the allocation of resources in the hospitals, which led to reduced staff motivation. In both hospitals, managers were unenthusiastic about participating in planning and budgeting meetings and often skipped them.

“It is not fair, it is not fair at all. I think they should at least allocate some money to me (physiotherapy department) like the other departments.” Middle-level manager, Hospital A

Another unintended behavior in both hospitals in response to the condition of resource scarcity was the use of historical allocations as one of the PSRA criteria. Managers in both hospitals felt that the severe scarcity of resources made it very difficult to objectively determine the relative allocation of resources to departments.

“How can you set priorities when there are no resources? We just give departments what we gave them last time, or add a bit more.” Senior manager Hospital B

Historical allocations had two effects. First, it entrenched the inequitable allocation of resources across departments and services. Second, both through entrenching inequitable allocation, and directly, it resulted in reduced motivation of hospital staff. Staff expressed frustration with always receiving the same allocations. A reinforcing feedback loop operated between historical budgeting and staff motivation (R2 in the CLD). Reduced staff motivation resulted in their disinterest in making revised budget proposals in subsequent budgeting cycles. Managers often presented the same proposals/requests as in previous periods. This reinforced the budgeting decision to allocate similar amounts as in previous periods, which further reduced the motivation of managers.

The case study hospitals also adopted short-termism in response to resource scarcity: hospitals’ PSRA activities focused on short term operational issues and neglected longer term strategic planning. Managers in both hospitals reported that even though the hospital had annual work plans and 5 year strategic plans, these plans had little relevance in the absence of resources to implement them. In both hospitals, there was therefore much more focus on meeting ad hoc needs that arose on a daily basis.

“It is management by putting off fires. Everyday there is a crisis that we have to sort out. Today there is no electricity, tomorrow the ambulance has broken down, such things. We try to plan but mostly we just try to keep the hospital running by dealing with the problems that we face day to day.” Middle level manager, Hospital A

### 3.3 Limited hospital autonomy and decision space

The decision space or autonomy experienced by the hospital is another factor that interacted with PSRA in the case study hospitals. Bossert (1998) has defined decision space as the range of effective choices and decision making authority that organizations have been given by central authorities. In examining the interaction between decision space and hospital PSRA, the case study hospitals, as CAS, are seen to respond to and adapt to conditions imposed by the larger system (the MOH) in which they are embedded. Specifically, both case study hospitals had adapted to situations where MOH regulations restricted their authority over certain decisions. For example, in both case hospitals, it was felt that the hospitals decision space with regard to their annual work plan (AWP) was limited. As part of the tangible software of systems and processes, hospital managers received guidance from the central MOH on what to include in the AWP including which list of health priorities to select from. However managers in both hospitals felt that this limited the hospitals’ autonomy in the sense that they could not select health problems that were not listed by the MOH.

“We don't feel like we contribute to the AWP [Annual work plan process]. It is like it is not ours. We just get these templates from the ministry and have to fill them according to instructions. The diseases are already listed there so just choose. Sometimes you want to include a priority but it is not in the list.” Middle level manager, Hospital A.

The limited flexibility with regard to the AWP resulted in a feeling that the process was not responsive to hospital needs. There was therefore a general feeling of lack of ownership and disinterest in the AWP process by the hospital managers, who saw it as a process that was conducted to meet government requirements but one that had little relevance to the hospital. This reduced staff motivation led to the non-implementation of hospital plans.

“I don't think anyone ever looks at the AWP or follows up to implement it. People feel like the ministry forces us to fill it but it is not relevant to us. We feel that it has ministry priorities but not hospital priorities.” Middle level manager, Hospital B

The reduced autonomy over hospital planning decisions led to frustration and reduced motivation among hospital managers and consistent with CAS interacted with other factors (resource scarcity, undesirable power dynamics in Hospital A, reduced managerial knowledge and skills, and weak monitoring and evaluation mechanisms) to contribute to the emergence of the “government culture” previously described which resulted in poor and/or rare of implementation of hospital plans. Poor implementation of plans interacted with the existence of a government culture in a reinforcing feedback loop (R3 in CLD). Because staff were disinterested in carrying out their duties and roles, plans were poorly and rarely implemented. The poor implementation of plans further promoted the government culture, which further worsened the implementation of plans.

Another emergent property of the case study hospitals to adapt to the constrained decision space over the AWP process was the development of a culture of “feigned compliance”. While managers appeared to, on paper, comply with all MOH guidelines, templates and timelines for the AWP process, in practice they either did not implement them or acted differently. Managers reported that they had to comply because it was an expectation of the MOH and also part of their performance contract. They however stated that this compliance was not translated into action because often these rules and guidelines were not in line with hospital priorities.

“That AWP we just prepare it because it is a ministry requirement. But in reality it is not followed or used in the hospital.” Middle level manager, Hospital B

Another example of the influence of decision space was the medicines selection process. Managers in both hospitals reported having low autonomy over what and how much to procure from KEMSA. The central MOH provided guidelines for what could be ordered by the hospitals in the form of an essential medicines list. In both hospitals, managers complained that this list was very restrictive and did not adequately meet the medicines needs of the hospital. To self organize in light of this situation, hospitals relied more on user fees to purchase medicines from local private providers, an avenue that was more flexible and responsive to hospital needs. As described in the previous section, this reliance on user fees resulted in unintended consequences.

### 3.4 Inadequate management and leadership capacity in the hospital

#### 3.4.1 Weak technical capacity of hospital managers for planning and budgeting

The limited capacity of hospital managers for planning and budgeting in both case study hospitals was regularly discussed.

“Most of the hospital management committee members do not have the training and skills in budgeting and planning. This makes the process of making the hospital annual work plan and budgets very difficult since they cannot even come up with simple budgets and plans for their departments.” Senior manager, Hospital A

This weakness in the hospital systems software of management, knowledge and skills, was attributed to a number of issues. First, hospital management was comprised of professionals who have received technical training in their profession but no management training.

“One day you are a dentist, the next day you are medical superintendent in charge of a big hospital. That is how it happens. You are sent here [the hospital] without any [management] training. All you have is your clinical training.” Senior manager, Hospital B

Professionals who take on the responsibility of managing professional work, professional colleagues and other staff have sometimes been referred to as hybrid managers (McGivern et al., 2015). It would be expected that these hybrid managers would be guided by colleagues who were management professionals such as the hospital accountant and the hospital administrative officer. However, it is widely recognized that the Kenyan public sector is characterized by uncooperative behavior among managers (working in silos). As outlined later, the power dynamics between the hospital accountant and administrator further impeded their corporation with other hospital managers. One of the manifestations of this reduced technical capacity for management was the lack of appreciation among hospital managers of the need to ration health care.

“The managers do not have budgeting and planning skills … it affects the budgeting process because they don't appreciate the process of budgeting and keep presenting unrealistic requests and do not understand why they can't always get what they ask for. Yet we don't have that money.” Senior manager, Hospital A

Failure to appreciate the need for rationing manifested itself in lengthy and inconclusive budgeting and planning meetings. It also led to the presentation of budgets that were out of touch with the reality of resource scarcity in the hospital, and non-alignment of budgets and plans. This lack of appreciation of the need to ration resources also contributed to the feeling of unfairness among hospital managers, which alongside other factors like resource scarcity led to reduced motivation (as described above) and the *emergence* of what was referred to several times by respondents as a *“government culture”* among hospital managers in the hospital system. The term “government culture” was used to refer to a situation where hospital staff seemed to lack a sense of commitment to (or seemed not to care about) what they were expected to do, to fail to take action or show initiative and to be generally lethargic towards their duties, including PSRA.

“In government nobody is serious about their work, people just show up to be seen but do not really care whether work is done or not.” Senior manager, Hospital A

The lack of technical competence in budgeting and planning, together with resource scarcity also contributed to the use of historical budgeting in hospital PSRA processes described above as an emergent property of the case hospitals.

“How can we use criteria to budget when they don't even know that they should be doing that? The only thing they know is last year's budget.” Senior manager, Hospital A

#### 3.4.2 The influence of the leadership characteristics of the medical superintendent

One of the striking differences between the two hospitals was the leadership and management capacity of the medical superintendent, who was the hospital chief executive. The medical superintendent in Hospital B appeared to be more motivated and committed to leadership and management roles. This aspect (hospital leader's motivation) of the hospitals' intangible software was therefore stronger in Hospital B compared to Hospital A, with the medical superintendent in Hospital A often unavailable to attend to administrative responsibilities resulting in complaints from other hospital managers.

“He doesn't have that time for management. Mostly he concentrates on what he likes, being a surgeon. I think the right person to be a medical superintendent is either a pediatrician or a gynecologist because they have time, they have time.” Middle level manager, Hospital A

The hospital superintendent was unavailable and less motivated to carry out their management and leadership roles due to individual as well as contextual factors. At the individual level, in what speaks to the professional identity of the medical superintendent in Hospital A, appeared to identify with his clinical responsibilities and hence place greater priority on this aspect of his job. He dedicated most of his time to clinical duties within the hospital and in private practice and neglected his managerial and leadership duties.

“I think the medical superintendent likes his theater work more than administration. If you talk to him about theater you can see that he is interested. But it is very difficult to find him to sort out administrative issues. I think he is not interested in that.” Middle level manager, Hospital A

At the contextual level, and a weakness in the systems’ tangible software, the appointment of hospital managers by the MOH was not consultative and did not take into account individual willingness and interest in taking up the position. The medical superintendent in Hospital A, was forced to be a manager despite expressing disinterest in the position. One had to become a hospital manager if assigned this responsibility by the central MOH:

“In government you don't choose to be a manager. You just wake up and you are told that you are now the medical superintendent of this or that hospital. You don't ask for it. They don't care whether you want it or not.” Middle level manager, Hospital A

Further, a weakness of the hospital system hardware, the hospital superintendent was the only surgeon in the hospital. Even though he had been appointed as a medical superintendent, with extensive leadership and management responsibilities, he had not been relieved of his clinical responsibilities and was still expected to run the surgical clinic and attend to all surgical cases. This splitting of time between management and clinical responsibilities is a weakness of the hospital tangible software of systems and processes.

The situation was quite different in Hospital B where the combination of both individual attributes and contextual factors resulted in better leadership. Here the medical superintendent's presence was felt in the hospital.

“We have a good medical superintendent. She is very dedicated. And she is also very helpful. She is always around to attend to issues and also for us to talk to her if we have a problem in our departments.” Middle level manager, Hospital B

At the individual level, and a strength in the hospitals’ intangible software, the medical superintendent in hospital B had a clear interest in management and clinical responsibilities and saw herself more as a manager than as a clinician (professional identity). At the contextual level, and a strength in the hospital hardware, her availability to carry out her management and leadership responsibilities was aided by the fact that she did not have a heavy clinical work burden. Given that the hospital had three other dentists, she had been relieved of her clinical responsibilities and focused fully on her management and leadership responsibilities. The medical superintendent in this hospital was very accessible to her staff.

This difference in leadership between the two case study hospitals depicts how the disposition and actions of an agent in a system, as well as system context can have far reaching effects and influence system dynamics. The unavailability of the medical superintendent in Hospital A meant that there was no one to manage the relationships and often varying interests of the different managers. This resulted in a further weakness in the hospital intangible software in the form of heightened power differences between managers that led a number to feel that there was unfairness in PSRA processes.

In Hospital A, the leadership vacuum at the top was unofficially filled by two other senior managers. Hospital managers in this hospital felt that the hospital administrator and the accountant had colluded to usurp the powers of the medical superintendent and used them to further their own interests. This contributed to feelings of unfairness and a sense of distrust among the managers.

“He is never there, so those two take over. And I told you about them, they only favor their departments and their friends. And since the medical superintendent is not there, nothing can be done about it. So most of us feel that especially the budgeting process is unfair.” Middle level manager, Hospital B

Compared to Hospital A, the presence and availability of the medical superintendent in Hospital B had contributed to a sense of fairness in the PSRA process. Managers reported that the medical superintendent was always at the budgeting and planning meetings and tried to ensure that every department got some allocation.

## 4 Discussion

The examination of factors influencing PRSA processes paints a picture of the case study hospitals as CAS. Aspects of both hospital systems hardware and software are seen to interact to influence PSRA in the case study hospitals. One of the key findings of these interactions is the important effect that hospital financing mechanisms have on PSRA practice. Against a background of scarce, unreliable and unpredictable supplies of resources both case study hospitals self-adjusted into organizations with a number of undesirable emergent properties including a “government culture” and revenue maximization behavior. This highlights the complex interactions among factors and agents within the hospital sub-system, and between the hospitals and the larger health system in which they are embedded, and the unpredictability of actions consistent with CAS theory (Ellis et al., 2011, Health Foundation, 2010, Schneider and Somers, 2006).

The emergence of revenue maximization was motivated by survival; the need for hospital managers to ensure that the hospital continued to operate despite resource scarcity. This is consistent with the feature of CAS as robust or resilient (Marion and Bacon, 2000). The case study hospitals, as sub-systems, appear to be loosely coupled to the larger national health system with regard to financing. The hospitals not only get funding from the central MOH, but also collect user fee revenues from hospital clients. While resource scarcity and central MOH funding delays pose a threat to the hospital operations, this loose coupling confers on them resilience by providing them with the flexibility to adapt by maximizing user fee revenues. As is seen in the case study hospitals however, resilience of systems does not always lead to desired outcomes. Systems can experience “mal-adaptation” to undesirable states (Marion and Bacon, 2000). The influence of hospital autonomy is another example of the interactions between a CAS and the larger system in which it is embedded. The significant influence that the central MOH had over hospital planning is shown in both case study hospitals to, among others, lead to the emergence of “feigned compliance” and “government culture”.

Another factor that interacted with PSRA in interesting ways is the state of leadership in the case study hospitals. While ‘hard’ leadership and management skills (e.g. budgeting and planning) were weak in both hospitals, the differences in the case study hospitals lay in the so-called ‘soft’ relational skills. For example, the PSRA process in Hospital B was more inclusive and deliberative, and perceived by hospital actors to be fair because the medical superintendent in this hospital reached out to different actors and “negotiated” with them to participate in the processes. The medical superintendent in this hospital also appeared to appreciate the power imbalance among actors, and sought to manage it by ensuring that everyone had an equal chance to contribute to decision making. This is in contrast to Hospital A, where the PSRA process was perceived by actors to be unfair and non-inclusive. The medical superintendent in this hospital made no effort to actively involve or empower the different actors in the hospital and hence the PSRA. This finding is significant given the observations in literature that often attention is given to hard skills while neglecting soft skills in leadership development initiatives (Daire et al., 2014).

A number of lessons can be drawn from the case hospitals in our study. First, it is imperative that public hospitals are adequately and sustainably resourced. Health systems should carry out assessments of the resource needs of public hospitals, both capital and recurrent, and mobilize adequate resources to enable these hospitals to function optimally. Second, policy makers need to rethink the financing mechanisms of public hospitals. For example, when cost-sharing arrangements are introduced in settings where government financing is inadequate and unreliable, hospitals are incentivized to align their operations to maximize user fee collections with undesirable equity implications. Also, waiver policies, while well intended, are often counter-productive if not accompanied by revenue compensations to facilities.

A third lesson is that both hard and soft management and leadership capacity should be strengthened in public hospitals. While in-service leadership and management training has been introduced in a number of developing country health systems, it is imperative that such programmes are adequately scaled up to cover a critical mass of the target group. Further, academic institutions of higher learning should introduce and integrate leadership and management training for pre-service health workers. This will not only increase their competence in these much needed skills, but also perhaps influence their professional identities to attach importance to management and leadership roles. However, unlike hard leadership competencies, it is unlikely that soft skills will be developed in managers by the formal “classroom type” leadership and management trainings (Daire et al., 2014). These skills are best learnt through doing, reviewing the situation carefully and considering how the learning can be built on for next time (Horner, 2002). One approach that has been proposed as a tool for the development of soft leadership skills is action learning (Daire et al., 2014). Action learning programmes are typically tailored to focus on the issues facing the organization and combine formal training with on the job mentoring (Daire et al., 2014); assignments and reflections from work experiences to achieve learning are incorporated. Another approach that has been promoted as a tool for leadership development is coaching (Daire et al., 2014). Coaching has been defined as an interactive process between a coach and client that helps the client improve, learn something, or take performance to the next level (Daire et al., 2014). During the coaching process, the coach applies a range of behavioral techniques and methods to help the client achieve a mutually identified set of goals to improve their professional performance, and consequently to improve the effectiveness of the clients’ organization within a formally defined coaching agreement (Kilburg, 2000).

The fourth lesson from our case study hospitals is that to improve the commitment and performance of hospital managers, measures should be taken to maintain motivation. While causes of motivation will vary across different contexts, one determinant of motivation that emerged from our study was the autonomy of health workers to choose whether or not to take up leadership and management responsibilities. Forcing health workers to be managers results in lack of commitment and motivation, and potentially removes competent staff from often much needed clinical roles. Also, health workers who choose to take on managerial responsibilities should be recognized and compensated for this, and those who take up full time management and leadership responsibilities should be relieved of some or all of their clinical responsibilities. Expecting managers to continue to perform all of their previous professional duties after taking up management responsibilities is setting them up for failure. It is important that hospital managers are given significant autonomy to make PSRA decisions. Autonomy empowers managers, promotes a sense of ownership of decisions and motivates managers to discharge their responsibilities.

In conclusion, it is imperative that the design and implementation of these interventions recognize that hospitals are CAS. In designing interventions, policy makers should proactively think about and anticipate the likely effects - positive or negative - of policies on the various components of the system as well as the full range of actors and stakeholders (Agyepong et al., 2012, Kwamie et al., 2014, Prashanth et al., 2014, Savigny and Adam, 2009). Further, within a complexity paradigm, interventions should focus on creating conditions that enable organizational effectiveness by fostering the conditions that allow desirable emergent future states by feeding the natural, bottom-up dynamics of emergence, innovation, and fitness, rather than determining or guiding effectiveness (Ford, 2009, Marion and Uhl-Bienb, 2001). In other words, intervening in CAS is about fostering productive emergence rather than simple, mechanistic, cause and effect type solutions.

## Figures and Tables

**Table 1:** Characteristics of case study hospitals.

Characteristic	Hospital A	Hospital B
Estimated Annual outpatient visits	60,000	60,000
Estimated Annual inpatient admissions	8000	5000
Estimated Annual monetary budget (USD)	450,000	280,000
Number of staff	234	236
Number of beds	183	166

**Table 2:** Number of participants selected in each hospital under each category.

National-level key informants	5	
	Hospital A	Hospital B
Senior managers	6	6
Mid-level managers	22	19
Front-line practitioners	7	8
Hospital sub-total	35	32
**Study total**	**72**	
